# Supplementary material for: Molecular Mechanism of Action of RORγt Agonists and Inverse Agonists: Insights from Molecular Dynamics Simulation
Source: Molecules. 2018 Dec 3;23(12):3181. doi: 10.3390/molecules23123181 (PMC6321388; doi:10.3390/molecules23123181)
Supplement: Supplementary file 1 [file molecules-23-03181-s001.zip › molecules-384611-supplementary/sp/molecules-384611-sp-for final.pdf]

molecules

Molecular Mechanism of Action of RORγt Agonists and Inverse Agonists: Insights from Molecular Dynamics Simulation

Nannan Sun †, Congmin Yuan †, Xiaojun Ma, Yonghui Wang, Xianfeng Gu * and Wei Fu *

Department of Medicinal Chemistry and Key Laboratory of Smart Drug Delivery, Ministry of Education, School of Pharmacy, Fudan University, Shanghai 201203, China; nysnn@126.com (N.S.); 16211030013@fudan.edu.cn (C.Y.); scuma@foxmail.com (X.M.); yonghuiwang@fudan.edu.cn (Y.W.)

* Correspondence: xfgu@fudan.edu.cn (X.G.); wfu@fudan.edu.cn (W.F.); Tel: +86-21-5198-0112 (X.G.); +86-21-5198-0026 (W.F.)

† These authors contributed equally to this paper.

Supporting information

1. Compounds identification

General. All the reagents were purchased from commercial suppliers and used as received. Purity and characterization of compounds were established by a combination of LC–MS and NMR. ^{1}H NMR spectra were recorded with a 400 MHz Varian spectrometer.The following abbreviations are used: br = broad signal, s = singlet, d = doublet, dd = doublet of doublets, t = triplet, q = quartet, and m = multiplet. Chemical shifts are expressed in ppm, and coupling constants (*J*) are expressed in Hz. All reactions were performed under nitrogen unless otherwise stated. High resolution mass spectrometry data were given by AB 5600+Q TOF. The purity of all the designed compounds was analyzed using an Agilent 1200 HPLC system with a G1311B quaternary pump, a G1329B ALS and a G4212B DAD detector. The HPLC method consisted of the following: Agilent C18 RP column (250 mm×4.6 mm, 5 lm); column temperature 25 °C; inject volume 2.0 lL; HPLC solvent H_2O (0.1% TFA)/CH_3OH = 60/40 (v/v); flow rate of 1.2 mL/min; detector wavelength of 254 nm.

1-(4-(1-(phenylsulfonyl)-1,2,3,4-tetrahydroquinolin-6-yl)piperazin-1-yl) ethan-1-one **(1)**
^{1}H NMR (400 MHz, DMSO) *δ* 7.65 (dd, *J* = 8.7, 4.4 Hz, 1H), 7.53 (d, *J* = 4.2 Hz, 4H), 7.49 (d, *J* = 9.0 Hz, 1H), 6.84 (dd, *J* = 9.0, 2.9 Hz, 1H), 6.63 (d, *J* = 2.8 Hz, 1H), 3.70 (dd, *J* = 6.7, 5.4 Hz, 2H), 3.56 (dd, *J* = 10.1, 5.1 Hz, 4H), 3.17 – 3.10 (m, 2H), 3.10 – 3.02 (m, 2H), 2.28 (t, *J* = 6.7 Hz, 2H), 2.04 (s, 3H), 1.48 (dd, *J* = 12.4, 6.3 Hz, 2H). LC-MS (ESI): t_R = 4.995 min, *m/z* = 400.0 $[M+H]^+$, *m/z* = 422.0 $[M+Na]^+$. HRMS (ESI^+) m/z calcd for $C_{21}H_{25}N_3O_3S$ $[M+H]^+$: 400.1689; found: 400.1688.

1-(4-(1-(benzylsulfonyl)-1,2,3,4-tetrahydroquinolin-6-yl)piperazin-1-yl)ethan-1-one **(2)**
^{1}H NMR (400 MHz, $CDCl_3$) *δ* 7.42 (d, *J* = 9.0 Hz, 1H), 7.38 – 7.28 (m, 3H), 7.21 (dd, *J* = 7.4, 1.6 Hz, 2H), 6.76 (d, *J* = 7.7 Hz, 1H), 6.68 (s, 1H), 4.33 (s, 2H), 3.79 (s, 2H), 3.64 (s, 2H), 3.52 – 3.34 (m, 2H), 3.22 – 2.98 (m, 4H), 2.64 (t, *J* = 6.6 Hz, 2H), 2.14 (s, 3H), 1.90 – 1.34 (m, 4H). LC-MS (ESI): t_R = 4.990 min, *m/z* = 413.9 $[M+H]^+$, *m/z* = 435.9 $[M+Na]^+$. HRMS (ESI^+) m/z calcd for $C_{22}H_{27}N_3O_3S$ $[M+H]^+$: 414.1846; found: 414.1835.

2. RORγt FRET assay

The assays were performed in an assay buffer consisting of 50 mM NaF, 50 mM 3-(*N*-morpholino)propanesulfonic acid, pH 7.4, 0.05 mM 3-[(3-cholamidopropyl) dimethylammonio] propanesulfonate, 0.1 mg/mL bovine serum albumin, and 10 mM dithiothreitol in 384-well plates. The total volume was 25 μL/well. The europium-labeled SRC1 solution was prepared by adding an appropriate amount of biotinylated SRC and europium labeled streptavidin into assay buffer, with final concentrations of 20 and 10 nM, respectively. The allophycocyanin (APC)-labeled-LBD solution

was prepared by adding an appropriate amount of biotinylated RORγt-LBD and APC-labeled streptavidin at final concentrations of 20 nM and 10 nM, respectively. After 15 min of incubation at room temperature, a 20-fold excess of biotin was added and incubated for 10 min at room temperature to block the remaining free streptavidin. Equal volumes of europium-labeled SRC and APC-labeled RORγt-LBD were then mixed and dispensed into 384-well assay plates at 25 μL volume/well. The 384-well assay plates had 100 μL of test compound in DMSO predispensed into each well. The plates were incubated for 1 h at room temperature and then read on Envision in LANCE mode configured for europeum-APC labels.

3. Mouse Th17 differentiation assay

CD4+ T cells were purified from mouse splenocytes using a commercial CD4+ T cell negative selection kit (Invitrogen). CD4+ T cells were skewed to Th17 cells by culturing cells in the presence of anti-CD3 (0.25 μg/mL, Bioxcel), anti-CD28 (1 μg/mL, Bioxcel), anti-IFN-γ (2 μg/mL, Bioxcel), anti-IL-4 (2 μg/mL, Bioxcel), TGF-β (5 ng/mL, Peprotech) and IL-6 (20 ng/mL, Peprotech) for 4 days before analysis. Compounds or DMSO control were added to the culture on day 0 of Th17 differentiation at indicated concentrations. Percentage of IL-17 production from CD4+ T cells were analyzed by intracellular staining followed by flow cytometry. Dose-response curves were plotted to determine half-maximal inhibitory concentrations (IC50) for the compounds using the GraphPad Prism 5 (GraphPad Software, San Diego CA, USA).

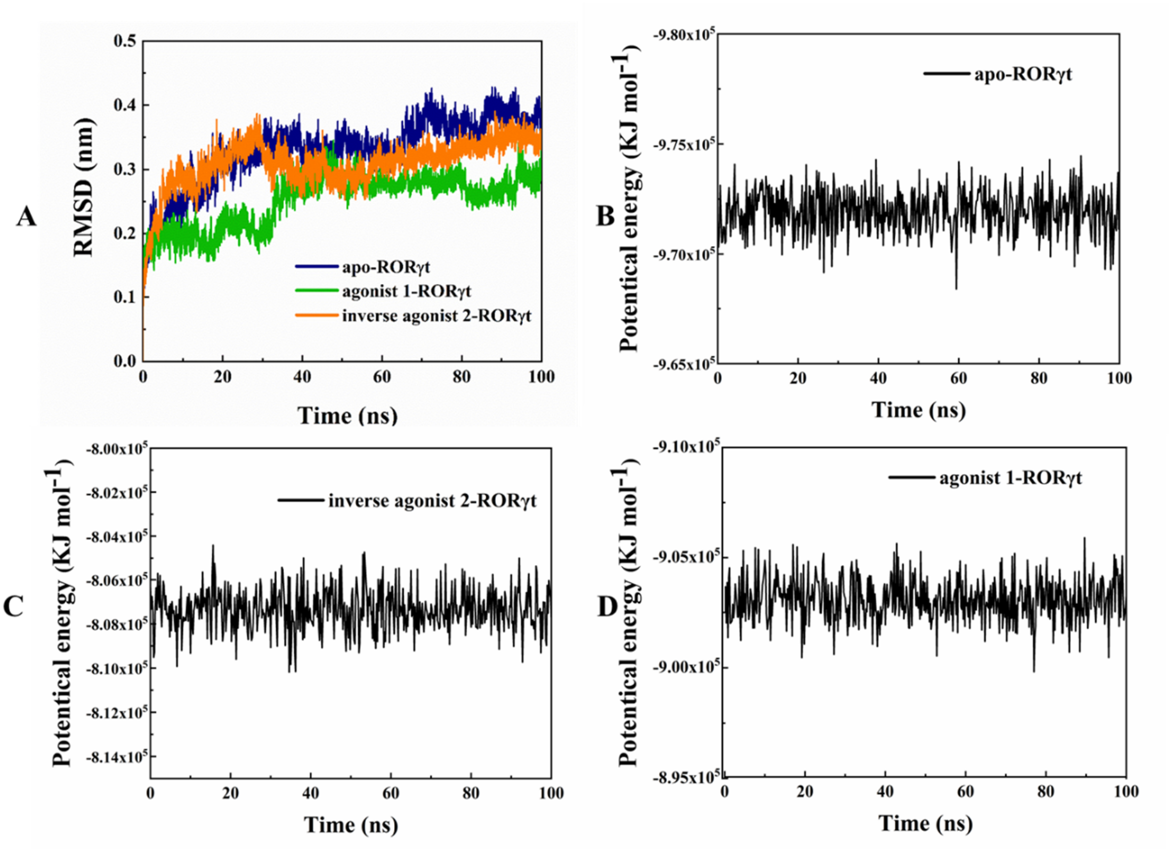

Figure S1. RMSD and potential energy. A, RMSD plot of backbone of RORγt in apo system (blue), agonist system (orange) and inverse agonist system (greem) systems. B, Plot of the potential energy of apo-RORγt system. C, Plot of the potential energy of inverse agonist-RORγt system systems. D, Plot of the potential energy of agonist-RORγt system.

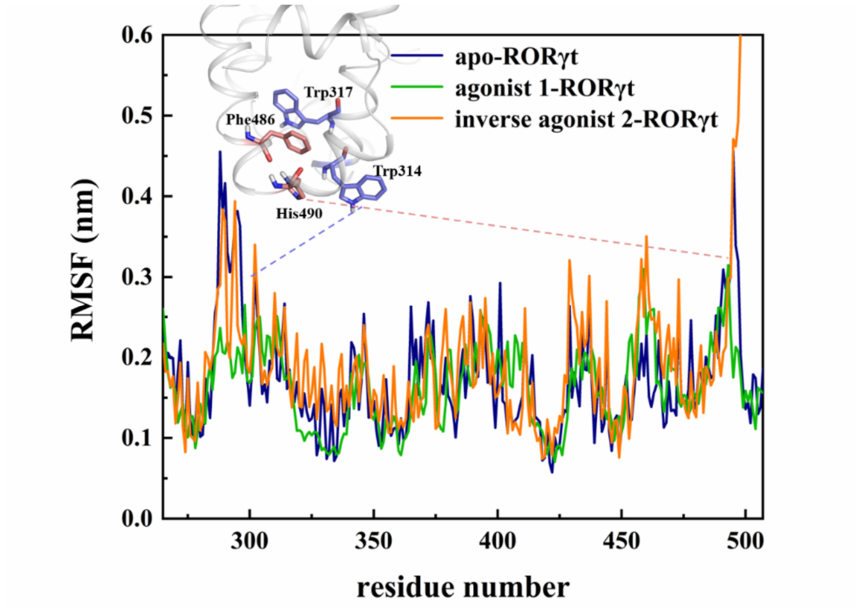

Figure S2. The RMSF plots of apo system (blue), agonist system (orange) and inverse agonist system (green) systems.

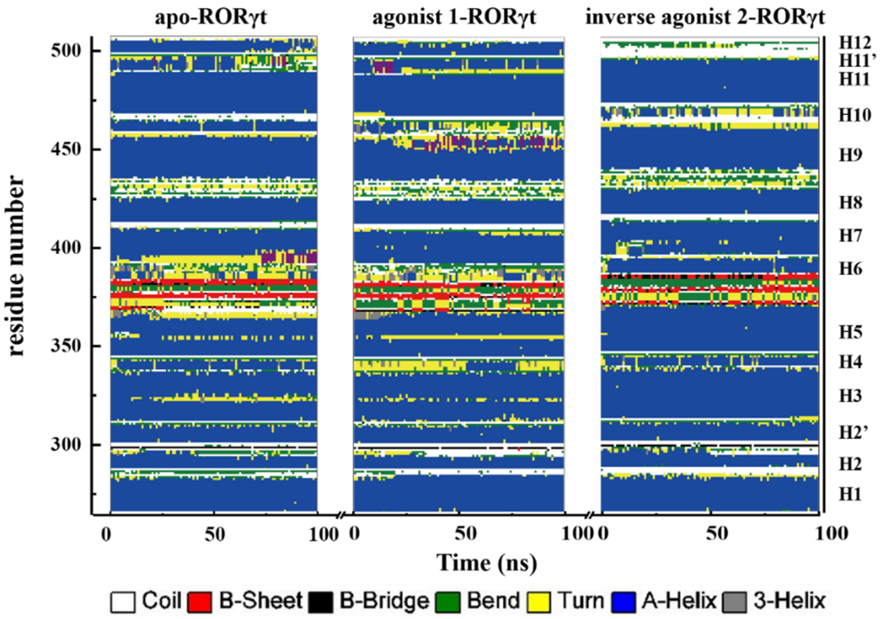

Figure S3. secondary structure analysis and representative conformations of RORγt-LBD as observed from apo-, agonist- and inverse agonist-RORγt system.

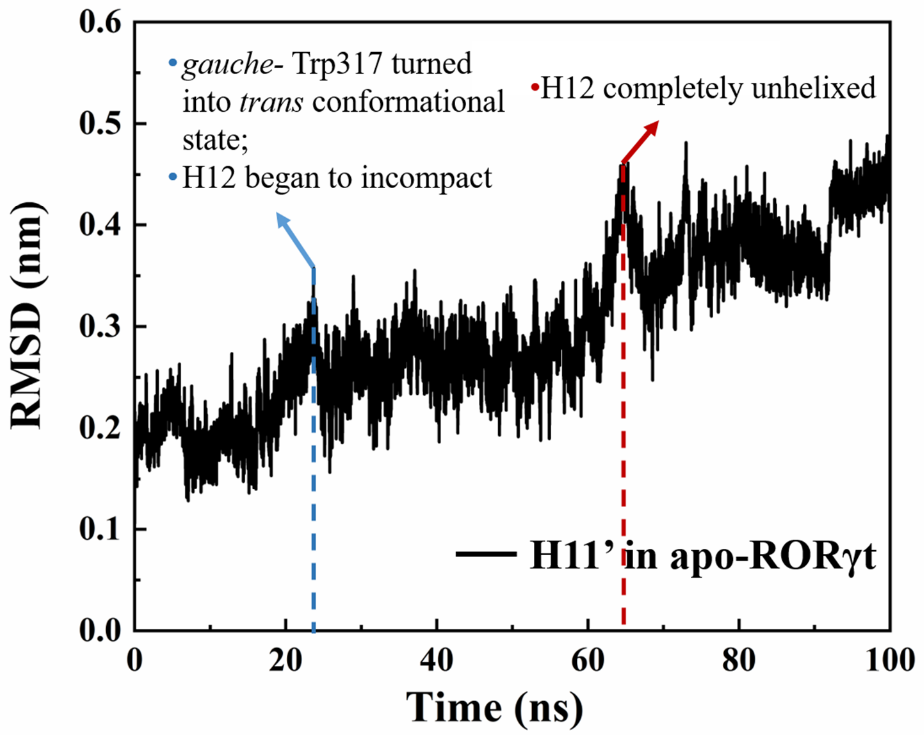

Figure S4. A, RMSD plot of backbone of H11′ in apo-RORγt system.

Movie S1. A video of molecular dynamics (MD) of the apo-RORγt system. Residues in the big hydrophobic cluster (Phe506, Tyr502, His479, Phe486, Trp317, Trp314 and His490) is shown in sticks. The red color stands for H12, orange for H11′, yellow for H11 and the green for H3.

Movie S2. A video of molecular dynamics (MD) of the agonist **1**-RORγt system. Agonist **1** and residues in the big hydrophobic cluster (Phe506, Tyr502, His479, Phe486, Trp317, Trp314 and His490) is shown in sticks. The red color stands for H12, orange for H11′, yellow for H11 and the green for H3.

Movie S3. A video of molecular dynamics (MD) of the inverse agonist **2**-RORγt system. Inverse agonist **2** and residues in the big hydrophobic cluster (His479, Phe486, Trp317, Trp314 and His490) is shown in sticks. The orange color stands for H11′, yellow for H11 and the green for H3.

Movie S4. A video of the MOA of RORγt agonism and inverse agonism.
